# Effects of Cycle Ergometer Exercise on Functional Capacity and Hospitalization Time of Patients After Open-Heart Surgery: A Systematic Review and Meta-Analysis

**DOI:** 10.21470/1678-9741-2024-0437

**Published:** 2026-06-08

**Authors:** Monielly Simas, Aline Almeida Gulart, Vicente Paulo Ponte Souza Filho, Katerine Cristhine Cani, Mariana Lanzoni Campos, Darlan Laurício Matte

**Affiliations:** 1 Postgraduate Program in Physiotherapy, Universidade do Estado de Santa Catarina, Florianópolis, Santa Catarina, Brazil; 2 Physical Therapy Department, Universidade do Estado de Santa Catarina, Florianópolis, Santa Catarina, Brazil; 3 Postgraduate Program in Human Movement Sciences, Universidade do Estado de Santa Catarina, Florianópolis, Santa Catarina, Brazil; 4 Multiprofessional Unit, Hospital Universitário da Universidade Federal de Santa Catarina, Florianópolis, Santa Catarina, Brazil

**Keywords:** Cardiac Rehabilitation, Hospitalization, Thoracic Surgery, Exercise.

## Abstract

**Introduction:**

Cycle ergometer in the postoperative period of open-heart surgery is a safe and economical exercise option. However, its specific effects, whether or not associated with conventional physiotherapy, are not well established in current literature. The objective of this study was to evaluate the effects of cycle ergometer exercise associated or not with conventional physical therapy, compared with only conventional physical therapy, on functional capacity, hospitalization time, peripheral muscle strength, and pulmonary complications of patients after open-heart surgery.

**Methods:**

MEDLINE, Cumulative Index to Nursing & Allied Health Literature, Latin American and Caribbean Health Sciences Literature, Web of Science, Scopus, Embase, Physiotherapy Evidence Database, and Cochrane Library were searched; manual searches were also conducted in the references of the included studies. Randomized controlled trials that analyzed the effects of cycle ergometer exercise associated or not with conventional physical therapy compared with only conventional physical therapy in adult patients after an open-heart surgery were included. Methodological quality was assessed by Cochrane risk-of-bias tool, and the meta-analysis was undertaken using RevMan 5.3.

**Results:**

Mean difference in the six-minute walk test (31 meters, 95% confidence interval [CI]: 1.59 to 60.3 meters, P = 0.04) was higher and in intensive care unit stay was lower (-0.5 days, 95% CI: -0.86 to -0.14 days, P = 0.007) in the intervention group. The total hospitalization time (-0.18 days, 95% CI: -0.73 to 0.38 days, P = 0.53) didn’t change between groups.

**Conclusion:**

Cycle ergometer exercises improved functional capacity but with no clinically relevant effects on hospitalization time after open-heart surgeries.

## INTRODUCTION

**Table:** 

Abbreviations, Acronyms & Symbols				
6MWT	= Six-minute walk test		IV	= Inverse variance
ACSM	= American College of Sports Medicine		LILACS	= Latin American and Caribbean Health Sciences Literature
CG	= Control group		LL	= Lower limbs
CI	= Confidence interval		MRS	= Myocardial revascularization surgery
CINAHL	= Cumulative Index to Nursing & Allied Health Literature		PEDro	= Physiotherapy Evidence Database
CPAP	= Continuous positive airway pressure		PO	= Postoperative
CR	= Cardiac rehabilitation		RPE	= Rate of perceived exertion
HR	= Heart rate		SpO₂	= Pulse oxygen saturation
ICU	= Intensive care unit		UL	= Upper limbs
IG	= Intervention group		VRS	= Valve replacement surgery

Cardiovascular diseases are the leading cause of death worldwide^[1]^. Surgery (*e.g.*, myocardial revascularization surgery [MRS] and valve replacement surgery [VRS]) may be indicated when hemodynamic changes or severe symptoms (or both) occur^[2,3]^. Despite technological advances, postoperative (PO) complications (*e.g.*, immobilism^[4-6]^, pain, cardiopulmonary bypass^[7]^, and prolonged mechanical ventilation^[8]^) are common and increase surgical morbidity and mortality^[9]^. These conditions also reduce functional capacity, which may be recovered by a four-phase cardiac rehabilitation (CR)^[10-12]^.

Phase I of CR begins after the patient achieves clinical stability and comprises low-intensity exercises^[13]^ seeking early mobilization^[14]^. These exercises include postural changes (i.e., sitting at the bedside or rolling on the bed), active training of upper and lower limbs, ambulation, and ascent and descent of steps performed progressively^[15,16]^. However, the protocol prescription (exercise type, intensity, duration, and progression) is not standardized, possibly impacting the absence of clinically relevant results. The difficulty in monitoring vital signs and exercise intensity^[17]^, especially during ambulation, may contribute to this issue. In addition, few intensive care units (ICU) have room for ambulation. Thus, the cycle ergometer can provide passive, active, and resistance training^[17,18]^. This intervention is a viable aerobic exercise in phase I of CR since it simplifies prescription and intensity monitoring.

Previous studies showed that cycle ergometer is a safe, low-intensity exercise for patients hospitalized after open-heart surgeries^[17,19]^. However, an adequate protocol for cycle ergometers is still being determined, and it is unclear whether its inclusion in the conventional physical therapy protocol would be effective.

Thus, this systematic review aimed to evaluate the effects of cycle ergometer exercise associated or not with conventional physical therapy, compared with only conventional physical therapy, on functional capacity and hospitalization time of patients after open-heart surgery.

## METHODS

This systematic review was conducted according to the Preferred Reporting Items of Systematic Reviews and Meta-Analyses statement^[20]^ and registered in the International Prospective Register of Systematic Reviews database (CRD42021265997).

### Literature Search and Screening

Searches were conducted in September 2022 in the following electronic databases: MEDLINE, Cumulative Index to Nursing & Allied Health Literature (or CINAHL), Latin American and Caribbean Health Sciences Literature (or LILACS - BIREME), Web of Science, Scopus, Embase, Physiotherapy Evidence Database (or PEDro), and Cochrane Library; manual searches were also conducted in the references of the included studies. The search strategy encompassed the keywords "cardiac surgery" and ("cycle ergometer", "early mobilization", "exercise," or "physiotherapy”) and the Boolean operators (AND and OR); no language or year restrictions were applied. The detailed strategy is described in Table 1. Two independent evaluators selected the studies. The order of evaluation was the title, abstract, and full text. Any disagreements in these steps were solved by consensus, and a third evaluator decided the eligibility when needed. The initial screening of titles, abstracts, and full texts was performed using the Rayyan® software.

**Table 1:** Search strategy.

Database	Search Strategy
MEDLINE	((Exercise Therapy[MeSH Terms]) OR (Rehabilitation[MeSH Terms]) OR (Early Ambulation[MeSH Terms]) OR (Physical Therapy Modalities[MeSH Terms]) OR (Exercise[MeSH Terms]) OR (Resistance Training[MeSH Terms]) OR (Rehabilitation[MeSH Terms]) OR (Cardiac Rehabilitation[MeSH Terms]) OR (Cycle ergomet^*^[Text Word]) OR (Bicycle ergomet^*^[Text Word]) OR (early mobili^*^[Text Word]) OR (physiotherapy[Text Word]) OR (physical therapy[Text Word])) AND ((Cardiac Surgical Procedures[MeSH Terms]) OR (Coronary Artery Bypass[MeSH Terms]) OR (Myocardial Revascularization[MeSH Terms]) OR (Cardiac Valve Annuloplasty[MeSH Terms]) OR (Heart Bypass, Left[MeSH Terms]) OR (Heart Valve Prosthesis Implantation[MeSH Terms]) OR (Heart Valve Prosthesis[MeSH Terms]) OR ((cardiac[Text Word] OR heart[Text Word] OR cardiovascular[Text Word] OR coronary[Text Word] OR myocardial[Text Word] OR aortic valv^*^[Text Word] OR mitral valv^*^[Text Word] OR tricuspid valv^*^[Text Word] OR pulmonary valv^*^[Text Word] OR bypass) N1 (operat^*^[Text Word] OR surg^*^[Text Word] OR procedure^*^[Text Word])) OR (CABG[Text Word] OR cardiac valvuloplasty[Text Word] OR heart valvuloplasty[Text Word] OR aortic valvuloplasty[Text Word] OR aortic valvotomy[Text Word] OR mitral valvuloplasty[Text Word] OR tricuspid valvuloplasty[Text Word]) OR ((cardiac[Text Word] OR heart[Text Word] OR cardiovascular[Text Word] OR coronary[Text Word] OR myocardium[Text Word] OR myocardial[Text Word] OR aortocoronary[Text Word] OR cardiopulmonary) N1 (bypass[Text Word] OR anastomosis[Text Word] OR revascularization[Text Word])) OR ((cardiac valv^*^[Text Word] OR heart valv^*^[Text Word] OR aortic valv^*^[Text Word] OR mitral valv^*^[Text Word] OR tricuspid valv^*^[Text Word] OR pulmonary valv^*^) N1 (implant^*^[Text Word] OR reimplant^*^[Text Word] OR replac^*^[Text Word] OR repair^*^[Text Word] OR reconstruct^*^[Text Word] OR artificial^*^[Text Word] OR prosthe^*^[Text Word] OR valvotom^*^[Text Word] OR valvulotom^*^[Text Word] OR annuloplast^*^[Text Word] OR valvuloplast^*^[Text Word] OR commissurotom^*^[Text Word])))
CINAHL - EBSCO	( SU Exercise Therapy OR SU Rehabilitation OR SU Early Ambulation OR SU Physical Therapy Modalities OR SU Exercise OR SU Resistance Training OR SU Rehabilitation OR SU Cardiac Rehabilitation OR TX ( Cycle ergomet^*^ OR Bicycle ergomet^*^ OR early mobili^*^ OR physiotherap^*^ OR physical therap^*^ )) AND ( SU Cardiac Surgical Procedures OR SU Coronary Artery Bypass OR SU Myocardial Revascularization OR SU Cardiac Valve Annuloplasty OR SU Heart Bypass, Left OR SU Heart Valve Prosthesis Implantation OR SU Heart Valve Prosthesis OR TX ((cardiac or heart or cardiovascular or coronary or myocardial or aortic valv^*^ or mitral valv^*^ or tricuspid valv^*^ or pulmonary valv^*^ or bypass) N1 (operat^*^ or surg^*^ or procedure^*^)) OR TX (CABG or cardiac valvuloplasty or heart valvuloplasty or aortic valvuloplasty or aortic valvotomy or mitral valvuloplasty or tricuspid valvuloplasty) OR TX ((cardiac valv^*^ or heart valv^*^ or aortic valv^*^ or mitral valv^*^ or tricuspid valv^*^ or pulmonary valv^*^) N1 (implant^*^ or reimplant^*^ or replac^*^ or repair^*^ or reconstruct^*^ or artificial^*^ or prosthe^*^ or valvotom^*^ or valvulotom^*^ or annuloplast^*^ or valvuloplast^*^ or commissurotom^*^))
LILACS	((Exercise Therapy) OR (Rehabilitation) OR (Early Ambulation) OR (Physical Therapy) OR (Exercise) OR (Resistance Training) OR (Cardiac Rehabilitation) OR (Cycle) OR (Bicycle) OR (early mobilisation) OR (early mobilization) OR (physiotherapy) OR (physical therapy)) AND ((Cardiac Surgical) OR (Coronary Artery Bypass) OR (Myocardial Revascularization) OR (Cardiac Valve Annuloplasty) OR (Heart Valve Prosthesis) OR (cardiac surgery) OR (heart surgery) OR (coronary surgery) OR (myocardial surgery) OR (valve surgery) OR (CABG) OR (valvuloplasty) OR (valvotomy))
EMBASE	('exercise therapy'/exp OR 'rehabilitation'/exp OR 'physical therapy modalities'/exp OR 'exercise'/exp OR 'cardiac rehabilitation'/exp OR 'cycle ergometer':ab,ti OR 'bicycle ergometer':ab,ti OR 'cycle ergometry':ab,ti OR 'bicycle ergometry':ab,ti OR 'early mobilisation':ab,ti OR 'early mobilization':ab,ti OR 'physiotherapy':ab,ti OR 'physical therapy':ab,ti) AND ('cardiac surgical procedures'/exp OR 'coronary artery bypass'/exp OR 'myocardial revascularization'/exp OR 'cardiac valve annuloplasty'/exp OR 'heart bypass, left'/exp OR 'heart valve prosthesis implantation'/exp OR 'heart valve prosthesis'/exp OR 'cardiac surgery':ab,ti OR 'heart surgery':ab,ti OR 'coronary surgery':ab,ti OR 'myocardial surgery':ab,ti OR 'valve surgery':ab,ti OR 'cabg':ab,ti OR 'valvuloplasty':ab,ti OR 'valvotomy':ab,ti)
Web of Science	#1: TS=("Exercise Therapy” OR “Rehabilitation” OR “Early Ambulation” OR “Physical Therapy Modalities” OR “Exercise” OR “Resistance Training” OR “Rehabilitation” OR “Cardiac Rehabilitation")
	#2: Ab=("cycle ergomet^*^” OR “bicycle ergomet^*^” OR “early mobili^*^” OR “physiotherapy” OR “physical therapy"))
	#3: #2 or #3
	#4: TS=("Cardiac Surgical Procedures” OR “Coronary Artery Bypass” OR “Myocardial Revascularization” OR “Cardiac Valve Annuloplasty” OR “Heart Bypass, Left” OR “Heart Valve Prosthesis Implantation” OR “Heart Valve Prosthesis")
	#5 AB=("cardiac surgery” OR “heart surgery” OR “coronary surgery” OR “myocardial surgery” OR “valve surgery” OR “cabg” OR “valvuloplasty” OR “valvotomy"))
	#6: #4 or #5
	#7: #3 and #6
SCOPUS	(TITLE-ABS-KEY ("Exercise") OR ("Rehabilitation") OR ("Cycle ergomet^*^") OR ("Bicycle ergomet^*^") OR ("early mobili^*^") ("physiotherapy") OR ("physical therapy") ) AND ( TITLE-ABS-KEY ("Cardiac Surgical") OR ("Coronary Artery Bypass") OR ("Myocardial Revascularization") OR ("Cardiac Valve Annuloplasty") OR ("Heart Valve Prosthesis") OR ("cardiac surgery") OR ("heart surgery") OR ("coronary surgery") OR ("myocardial surgery") OR ("valve surgery") OR ("CABG") OR ("valvuloplasty") OR ("valvotomy") )
Cochrane	#1: MeSH descriptor: [Exercise therapy] explode all trees
	#2: MeSH descriptor: [Rehabilitation] explode all trees
	#3: MeSH descriptor: [Physical therapy modalities] explode all trees
	#4: MeSH descriptor: [Exercise] explode all trees
	#5: MeSH descriptor: [Cardiac rehabilitation] explode all trees
	#6: (cycle ergomet^*^)
	#7: (bicycle ergomet^*^)
	#8: #1 or #2 or #3 or #4 or #5 or #6 or #7
	#9: MeSH descriptor: [Cardiac surgical procedures] explode all trees
	#10: MeSH descriptor: [Coronary artery bypass] explode all trees
	#11: MeSH descriptor: [Myocardial revascularization] explode all trees
	#12: MeSH descriptor: [Cardiac valve annuloplasty] explode all trees
	#13: MeSH descriptor: [Heart bypass, left] explode all trees
	#14: MeSH descriptor: [Heart valve prosthesis implantation] explode all trees
	#15: MeSH descriptor: [Heart valve prosthesis] explode all trees
	#16: (cardiac surgery)
	#17: (heart surgery)
	#18: (valve surgery)
	#19: #9 or #10 or #11 or #12 or #13 or #14 or #15 or #16 or #17 or #18
	#20: #8 and #19
PEDro	Strategy 1: 3326
	Title & abstract field: Exercise
	Subdiscipline: cardiothoracics
	Method: Clinical trial
	Terms were reached in association (“AND”)
	Strategy 2: 181
	Title & abstract field: cycle ergomet^*^
	Subdiscipline: cardiothoracics
	Method: Clinical trial
	Terms were reached in association (“AND”)
	Strategy 3: 78
	Title & abstract field: bicycle ergomet^*^
	Subdiscipline: cardiothoracics
	Method: Clinical trial
	Terms were reached in association (“AND”)
	Strategy 4: 90
	Title & abstract field: Early mobili^*^
	Subdiscipline: cardiothoracics
	Method: Clinical trial
	Terms were reached in association (“AND”)
	Strategy 5: 1482
	Title & abstract field: Rehabilitation
	Subdiscipline: cardiothoracics
	Method: Clinical trial
	Terms were reached in association (“AND”)
	Strategy 6: 348
	Title & abstract field: Physical therapy
	Subdiscipline: cardiothoracics
	Method: Clinical trial
	Terms were reached in association (“AND”)
	Strategy 7: 492
	Title & abstract field: Physiotherapy
	Subdiscipline: cardiothoracics
	Method: Clinical trial
	Terms were reached in association (“AND”)
CINAHL=Cumulative Index to Nursing & Allied Health Literature; LILACS=Latin American and Caribbean Health Sciences Literature; PEDro=Physiotherapy Evidence Database	

### Inclusion and Exclusion Criteria

Only randomized controlled trials were included.

### Patients

Patients over 18 years in the PO period of open-heart surgery were included.

### Interventions

The patients from the intervention group performed lower limb exercises in the ICU or wards using a portable cycle ergometer while sitting on the bed (supine position with elevated headboard), at the bedside, or in an armchair. This intervention was associated or not with conventional physical therapy.

The control group performed conventional physical therapy, defined as any active upper or lower limb exercises (*e.g.*, walking and exercises [resistance, free active, or isometric]), except the cycle ergometer. Studies that applied just breathing exercises, stretching, neuromuscular electrical stimulation, or passive mobilization were excluded.

### Outcomes

The primary outcomes were the hospitalization time in days (*i.e.*, total and ICU stay), functional capacity measured by the distance walked (in meters or % of predicted) in the six-minute walk test (6MWT), and score on functional scales.

The secondary outcomes were pulmonary complications *(i.e.*, atelectasis, pleural effusion, lung infections, respiratory failure, reintubation), peripheral muscle strength measured by the Medical Research Council score, quadriceps cross-sectional diameter, dynamometry, or handgrip strength.

### Data Extraction

Two independent evaluators extracted and plotted data in a spreadsheet (Microsoft Excel®). The extracted data encompassed the type of exercise, intensity, time or repetitions, daily and weekly frequency, and form of progression of intervention and control groups. In addition, the 6MWT data were used to analyze the functional capacity. Hospitalization time (days) corresponded to the total time and ICU stay.

### Risk of Bias Assessment

Two independent reviewers assessed the risk of bias using the Cochrane risk of bias tool. This tool comprises seven items that classify the risk of the study as low, high, or uncertain^[21]^. Any disagreements were solved by consensus, and a third evaluator was recruited when needed.

### Data Analysis

Meta-analyses were performed in the Review Manager (version 5.2). The random effect model was used due to the heterogeneity of exercise prescription between studies. Heterogeneity was assessed using The Chi-square test (α = 0.10) and (I2). Two studies reported data (6MWT^[22]^ and hospitalization time^[19]^) as median and interquartile range; authors were contacted and provided these data in mean and standard deviation for inclusion in the meta-analysis.

## RESULTS

### Literature Search and Screening

**Fig. 1:**
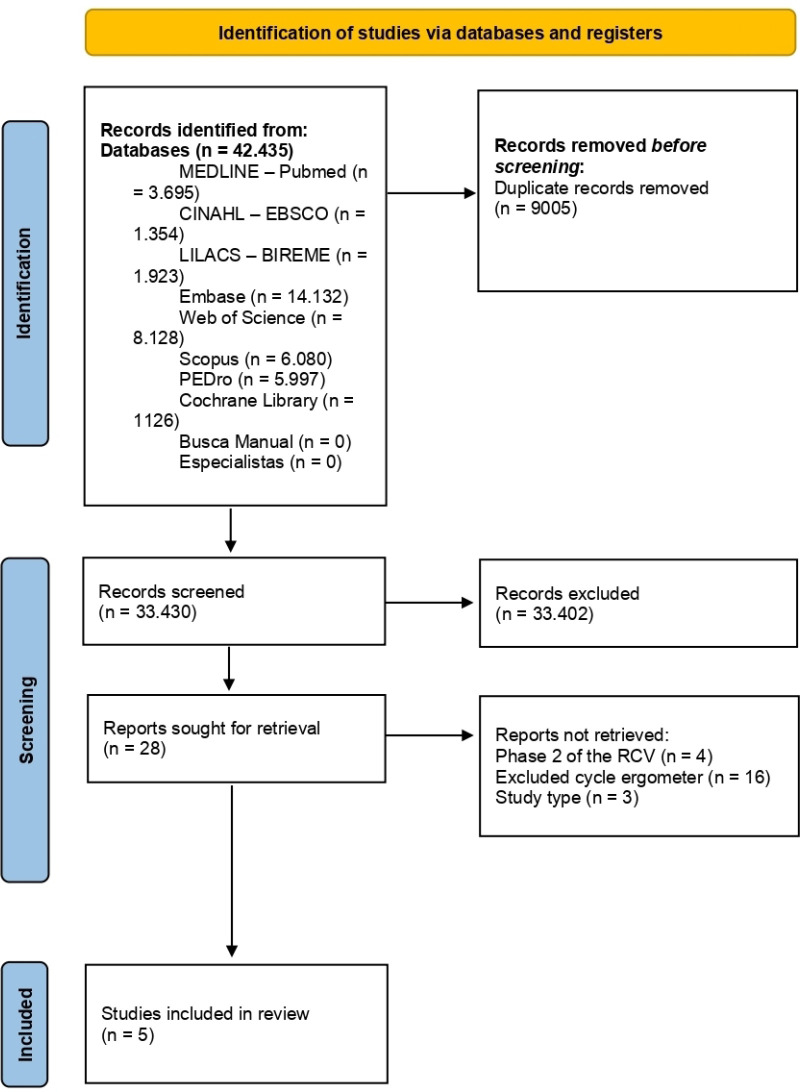
Flow diagram of studies assessed for eligibility. CINAHL=Cumulative Index to Nursing & Allied Health Literature; CR=cardiac rehabilitation; LILACS=Latin American and Caribbean Health Sciences Literature; PEDro=Physiotherapy Evidence Database.

A total of 42,435 studies were found in the initial screening; 9,005 duplicates were removed, and 28 studies were eligible for full evaluation. Then, 23 studies were excluded, and five were included in this review (Figure 1).

### Basic Information about the Included Trials

Data on patients, intervention, control group, outcomes, and intervention effects compared with the control group are reported in Table 2. Of the five studies included, four are Brazilian^[17,19,22,23]^ and one is Australian^[24]^.

**Table 2:** Characterization of studies.

Study	Participants	Control	Intervention	Outcomes
Trevisan et al. 2015	N = 24 CG = 10 / IG = 14 Male: CG = 80% / IG = 71% PO of MRS Average age: CG = 63.2 ± 11.1 years IG = 58.0 ± 8.1 years	3^rd^ PO day: changes in bed position and secretion removal techniques when necessary; 4^th^ - 7^th^ PO days: pulmonary reexpansion techniques, specific physical exercises and progressive walking; From the 5^th^ PO day: up and down the stairs with supervision. Frequency: 2 times a day; Duration: 20 min.	Similar to the CG protocol, except by replacing walking and stair exercises for cycle ergometer exercise since 3^rd^ PO day. Frequency: 2 times a day; Duration: 20 minutes. HR tolerated was up to 30 bpm above the resting HR.	*6MWT:* CG: 212 ± 69.6 m 3^rd^ PO *vs.* 250 ± 61.4 m 6^st^ PO or discharge (*P* < .001); IG: 208 ± 71.4 m preoperative 3^rd^ PO *vs.* 312 ± 80.6 m 6^rd^ PO or discharge (*P* < .001); *Comparison:* there was no significant difference between the groups.
Borges et al. 2016	N = 34 CG = 15 / IG = 19 Male: CG = 52.6% / IG = 80% PO of MRS Average age: CG = 62.8 ± 42.0 years IG = 62.5 ± 7.1 years	From 1^st^ PO day to hospital discharge: Secretion removal techniques, diaphragmatic breathing exercises, assisted and active exercises for UL and LL, and progressive walking. Frequency: 2 times a day in the ICU and 1 time a day in the ward until hospital discharge; Duration: Not clear.	Protocol similar to the CG, with an additional exercise with cycle ergometer, without load, initiating from 1^st^ PO day to hospital discharge. Frequency: 2 times a day in the ICU and 1 time a day in the ward until hospital discharge. Duration in the ICU: 5 minutes. Duration in the ward: 1^st^-2^nd^ PO days: 10 minutes. From 3^rd^ PO day to discharge: 20 min. Patients were instructed to pedal as fast as possible, maintaining the same pace used during the intervention period.	*6MWT:* CG: 320 (288 - 393 m) preoperative *vs.* 292 (237 - 336 m) PO (*P* = .01); IG: 364 (324 - 428 m) preoperative *vs.* 348 (301 - 414 m) PO (*P* = .06); *Comparison:* The distance in the 6MWT was kept in the IG but significantly decreased in CG patients. A significant difference was observed in the intergroup analysis of hospital discharge (*P* = .03). *ICU length of stay:* CG: 3.6 ± 1.4 days; IG: 3.2 ± 1.4 days; *Comparison:* there was no significant difference between the groups (3.4 ± 1.4 days; *P* = .31). *Hospital length of stay:* CG: 8.3 ± 2.2 days; IG: 8.1 ± 0.9 days; *Comparison:* there was no significant difference between the groups (8.2 ± 1.7 days; *P* = .53).
Gama Lordello et al. 2020	N = 228 CG = 117 / IG = 111 Male: CG = 57% / IG = 59% PO of MRS, VRS, and/or combined Average age: CG = 58.2 ± 12.9 years IG = 57.2 ± 13.2 years	From 6 h - 8 h post-extubation to ICU discharge: 10 active exercises repetitions for LL (straight leg raise, hip flexion-extension, knee flexion-extension, and ankle flexion-extension) and UL (diagonal movements). Frequency: 2 times a day; Duration: 10 min.	From 6 h - 8 h of extubation to ICU discharge: only the cycle ergometer was used for both LL and UL. The individuals were instructed to pedal continuously without any additional load. For LL, the headboard was declined to a 30° to provide better access to the pedals and prevent compensatory hip movements. For UL, individuals were positioned with the headboard elevated to 60° horizontally. After drain removal, physical activity progressed to standing, sitting in a chair and walking in the ICU hall. Frequency: 2 times a day; Duration: 10 min (5 min. for UL and 5 min. for LL).	*ICU length of stay:* CG: 2 (2 - 4 days). IG: 2 (2 - 3 days). *Comparison:* there was no significant difference between the groups (*P* = .09). *Hospital length of stay:* CG: 8 (7-14 days). IG: 8 (7-11 days). *Comparison:* there was no significant difference between the groups (P = .28).
Hirschhorn et al. 2012	N = 64 CG = 32 / IG = 32 Male: CG = 88.2% / IG = 70.4% PO of MRS Average age: CG = 65.9 ± 8.7 years IG = 65.3 ± 9.8 years	1^st^ and 2^nd^ PO days: motor and breathing exercises, transfers, and walking. From the 3^rd^ PO day until hospital discharge: walking progression (BORG Modified 3 - 4/10). Before discharge, the patient went up and down the stairs with supervision. Guidance was given about the exercise progression after the hospital discharge and forwarded referred to CR ambulatory. Frequency: 2 times a day; Duration: 10 min.	Protocol similar to the CG, except for from 3^rd^ PO day to discharge, walking was replaced by exercise using the cycle ergometer. Frequency: 2 times a day; Duration: 10 min. Patients were instructed to maintain the pace that results in 3 - 4/10 BORG rating.	*6MWT:* CG: 484 ± 86.0 m preoperative *vs*. 417 ± 86.0 m PO; IG: 450 ± 76.0 m preoperative *vs.* 401 ± 93.0 m PO; *Comparison:* both groups reduced the distance covered (*P* < .001), with no significant difference between the groups (*P* = .80). *Hospital length of stay:* CG: 7.7 ± 2.7 days / 7.0 (7 - 8 days); IG: 7.9 ± 1.7 / 7.0 (7 - 8.5 days); *Comparison:* there was no significant difference between the groups (*P* = .34).
Windmöller et al. 2020	N = 31 CG = 16 / IG = 15 Male: CG = 62.5% / IG = 80% PO of MRS Average age: CG = 62 ± 6.0 years IG = 57 ± 8.0 years	From immediate PO to hospital discharge: exercise program in seven progressive stages. The exercises began in bed progressing to sitting position with subsequent walking (initiating in 35 m, progressing to 200 m through the days) and up and down the stairs (starting with down 1 level to up and down 3 levels), plus stretches. In all stages, if necessary, CPAP was used. Frequency: 2 times a day; Duration: average of 25 min. HR tolerated was up to 30 bpm above resting HR.	Protocol similar to that of the CG, including the cycle ergometer and CPAP from the 2^nd^ to the 4^th^ PO day. On the 4^th^ PO day, the intervention was done in the morning and was measured in the afternoon. Frequency: 1 time a day (cycle ergometer + CPAP) + 2 times a day (conventional protocol); Duration: 20 min. on the 2^nd^ PO day and 30 min. on the other days. HR tolerated was up to 30 bpm of the resting value, SpO₂ up to 90% and BORG 2 - 3/10.	*6MWT:* CG: 290 ± 86.1 m preoperative *vs.* 181 ± 60.5 m PO (*P* < .001); IG: 264 ± 132.0 m preoperative *vs.* 216 ± 75.1 m PO (*P* =.11); *Comparison:* there was no significant difference between the groups in the preoperative (*P* = .52) and PO (*P* = .16) periods. *Length of stay in the ICU:* CG: 2.9 ± 0.7 days; IG: 2.5 ± 0.5 days; *Comparison:* there was no significant difference between the groups (*P* = .05). *Length of hospital stay:* CG: 7.3 ± 1.2 days; IG: 7.1 ± 1.1 days; *Comparison:* there was no significant difference between the groups (*P* = .69).
6MWT=six-minute walk test; CG=control group; CPAP=continuous positive airway pressure; CR=cardiac rehabilitation; HR=heart rate; ICU=intensive care unit; IG=intervention group; LL=lower limbs; MRS=myocardial revascularization surgery; PO=postoperative; SpO₂=pulse oxygen saturation; UL=upper limbs; VRS=valve replacement surgery Data was presented as median (interquartile range) and mean ± standard deviation				

The number of patients varied between 24 and 228, totaling 381 (190 in the control group and 191 in the intervention group). Four studies^[17,22-24]^ included only patients who underwent MRS and one^[19]^ who underwent MRS, VRS, or both combined. Patients included were aged over 50 years^[17]^, between 40 and 75 years^[23]^, and over 18 years^[19]^; two studies^[22,24]^ did not consider age as an inclusion criterion. Four studies^[17,19,22,24]^ excluded patients with orthopedic, neurological, and vascular limitations; one^[23]^ excluded only those with PO complications. Trevisan et al.^[17]^ also excluded patients who had complications (preoperative and PO) and were reintubated. Borges et al.^[22]^ excluded patients who underwent surgery and stayed more than ten days in the hospital. Gama Lordello et al.^[19]^ excluded patients who had difficulty understanding the study activities and those who discontinued the protocol in the ward to return to the ICU. Lastly, Hirschhorn et al.^[24]^ excluded planned concomitant surgery, emergency MRS, and non-English speakers.

### Intervention and Control Groups

Considering the interventions, one study^[19]^ performed cycle ergometer exercises associated with ambulation progression, and four^[17,22-24]^ added the cycle ergometer to the conventional physical therapy. In Windmöller et al.^[23]^, the intervention group used continuous positive airway pressure during cycle ergometer exercises; the control group used it only if needed. One study^[24]^ used a stationary bicycle^[24]^, while the others^[17,19,22,23]^ used a portable cycle ergometer. One study^[23]^ started the exercise on a cycle ergometer from the second PO day, two^[17,24]^ from the third, and two^[19,22]^ within 24 hours. Borges et al.^[22]^ started cycle ergometer exercises in the ICU, and Gama Lordello et al.^[19]^ performed them only in the ICU; three studies^[17,23,24]^ did not specify where the protocol started. The cycle ergometer exercises, in general, ranged between 10^[19,24]^ and 30 minutes^[17]^. Borges et al.^[22]^ prescribed exercises for five minutes in the ICU, and the time progressively increased to 20 minutes. All studies performed the exercises twice a day, except for Windmöller et al.^[23]^, who conducted the exercise on a cycle ergometer once a day.

The safety criteria, when reported, were the same for both groups. Trevisan et al.^[17]^ used a tolerated heart rate (HR) of 30 bpm above resting HR and signs of exercise intolerance. Hirschhorn et al.^[24]^ interrupted the exercise when HR was over 90% of the predicted maximum or if it fell 40 bpm in less than one minute. Gama Lordello et al.^[19]^ reported control and monitoring of vital signs but did not provide details on the considered parameters. Windmöller et al.^[23]^ interrupted the intervention if the patient presented fatigue, chest pain, dyspnea, cyanosis, pallor, tachycardia (> 120 bpm), bradycardia, complex arrhythmias, or hypotension. Borges et al.^[22]^ did not report an interruption criterion; however, regarding intensity control and exercise progression, they instructed patients to pedal as fast as possible, maintaining the pace during the intervention period and progressing over time. Gama Lordello et al.^[19]^ instructed the patients to pedal continuously. Hirschhorn et al.^[24]^ instructed pedaling in a rate of perceived exertion (RPE) of 3 to 4 on the Borg scale (*i.e.*, moderate to somewhat strong) without progression. Windmöller et al.^[23]^ used as a criterion an increase of up to 30 bpm above resting HR, RPE of 2 to 3, and minimum peripheral oxygen saturation of 90% without oxygen supplementation. No study reported adding load to cycle ergometer exercise.

### Outcomes

Two studies^[22,24]^ assessed the outcomes before surgery and at discharge; one study^[19]^ in the PO period (second, third, and fourth day); one study^[23]^ before surgery and four days after, and one study^[17]^ one and three days after surgery, with a reassessment on the sixth day or at discharge. Three meta-analyses are conducted for the outcomes: functional capacity, ICU stay, and hospital stay. Sensitivity analysis was not performed because all meta-analyses were homogeneous (*P* ≥.1 and I^^2^^ ≤ 25%). Peripheral muscle strength and pulmonary complications were not assessed in the included studies. The results of each study are reported in Table 2.

### Functional Capacity

**Fig. 2:**
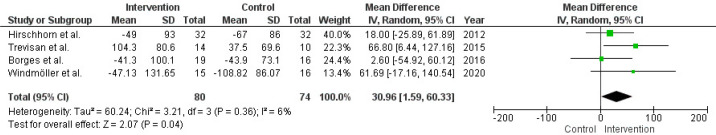
Forest plot of comparison: mean difference of 6-minute walk test (m). CI=confidence interval; IV=inverse variance; SD=standard deviation.

Four studies^[17,22-24]^ assessed the functional capacity using the 6MWT (Figure 2). The mean difference in 6MWT (final 6MWT - initial 6MWT) was higher in the intervention group than in the control group (31 meters, 95% confidence interval [CI]: 1.59 to 60.30 meters, *P* = 0.040; I^^2^^ = 6%, *P* = 0.360).

### Hospitalization Time

**Fig. 3:**
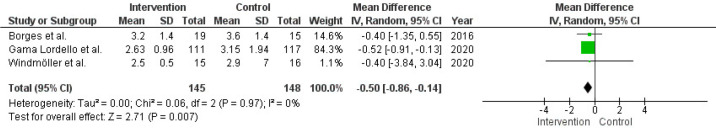
Forest plot of comparison: length of intensive care unit stay (days). CI=confidence interval; IV=inverse variance; SD=standard deviation.

**Fig. 4:**
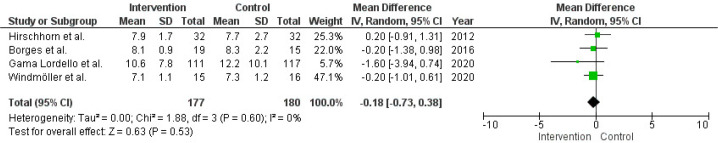
Forest plot of comparison: length of hospital stay (days). CI=confidence interval; IV=inverse variance; SD=standard deviation.

Three studies^[19,22,23]^ reported the ICU stay (Figure 3); the mean difference was lower in the intervention group than in the control group (-0.5 days, 95% CI: -0.86 to -0.14 days, *P* = 0.007; I^2^ = 0%, *P* = 0.970). Four studies^[17,22-24]^ evaluated the total hospitalization time (Figure 4), which was not significantly different between groups (-0.18 days, 95% CI: -0.73 to 0.38 days, *P* = 0.53; I^2^ = 0%, P = 0.600).

### Risk of Bias

**Fig. 5:**
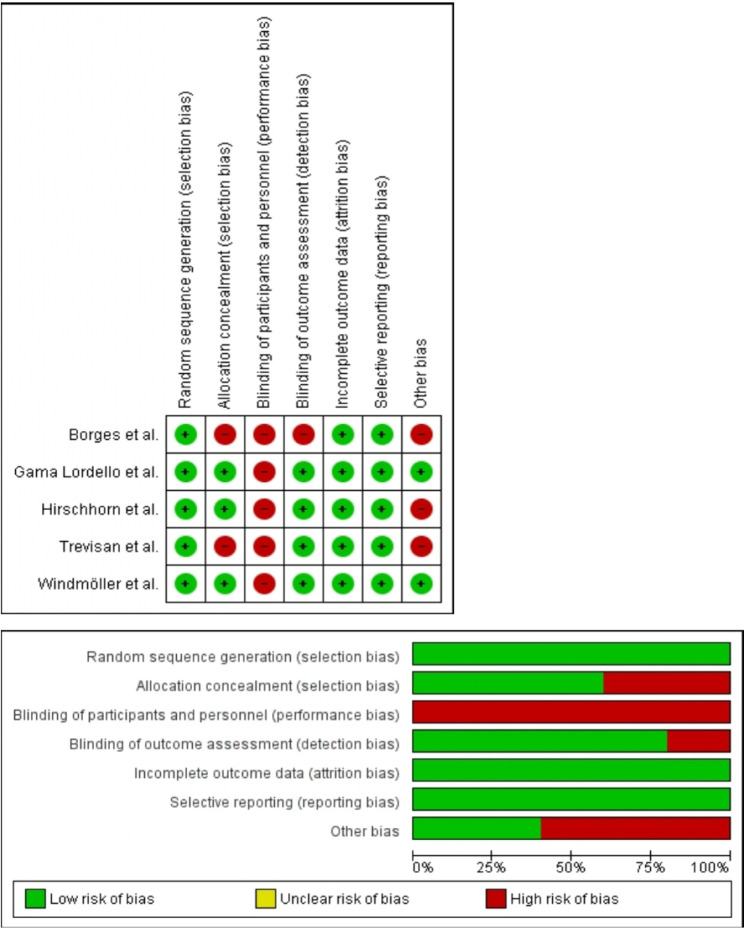
Risk of bias.

All studies^[17,19,22,24]^ showed a low risk of bias regarding random sequence generation, incomplete outcome data, and selective reporting; however, they showed a high risk regarding blinding patients and personnel. Two studies^[17,22]^ presented a high risk of bias for allocation concealment, and one of these^[22]^ also had a high risk for blinding of outcome assessment. Moreover, only two studies^[19,23]^ demonstrated a low risk for other biases. Two studies had as bias the lack of sample calculation^[22,24]^, a sample calculation not fulfilled^[17]^, and the age difference between the groups in one of the studies^[17]^. The risk of bias of the included studies is shown in Figure 5.

## DISCUSSION

The present study suggests that adding cycle ergometer exercise to conventional physical therapy improve functional capacity and ICU stay in patients hospitalized after open-heart surgery.

Patients with cardiovascular diseases with an indication for surgery often have previous impairment of functional capacity^[25]^, which may worsen in the PO period^[26]^ due to bed immobilization, pulmonary complications^[27]^, pain^[28]^, and fatigue^[29]^. Kanejima et al.^[15]^ demonstrated in a meta-analysis that patients who received early mobilization after an open-heart surgery performed better in the 6MWT at discharge than the control group (mean difference = 54 meters; 95% CI: 31.1 to 76.9 meters), suggesting that exercises during hospitalization time improve functional capacity. The present study found that the mean difference of functional capacity was greater than the minimal clinically important difference observed in patients with chronic heart failure and coronary artery disease (14.0 to 30.5 meters)^[30,31]^, and almost reached the minimal detectable change of patients in the PO period of coronary artery bypass graft surgery (36.1 meters)^[32]^. This finding suggests that the cycle ergometer exercise added to a conventional physical therapy protocol may significantly improve the 6MWT performance and that this improvement is clinically relevant.

Functional capacity assessed by 6MWT was associated with important outcomes in the PO period of cardiac surgeries. Patients who walk < 300 meters in the 6MWT have lower survival, greater PO complications^[25,33]^, and the inability to perform it indicates clinical severity and worse outcomes^[34]^. Therefore, interventions for improving functional capacity are crucial for this population. Although early mobilization improves functional capacity, this intervention did not influence the hospitalization time^[35]^. In the present study, adding cycle ergometer exercise to conventional physical therapy reduced the ICU stay, but the total hospitalization time did not change. The reduced ICU stay is important because the ICU is costly for hospitals^[36]^. However, the present study found a mean difference of 0.5 days, which was not clinically relevant. It is important to highlight that ICU stay can be affected by other factors unrelated to the functional status of the patient, such as PO complications, associated comorbidities, and infections^[37]^.

According to the American College of Sports Medicine (ACSM)^[38]^, proper exercise prescription should individually define the type of exercise, intensity, frequency, duration, form of progression, and volume. Intensity prescription should be based on parameters (*e.g.*, HR, oxygen consumption, or metabolic equivalents)^[38]^. In the present review, only four studies^[19,22-24]^ described intensity control parameters and all were subjective (Borg scale^[23,24]^ and verbal orientations^[19,22]^). Hirschhorn et al.^[24]^ and Windmöller et al.^[23]^ used the RPE (modified Borg scale) of 3 to 4 and 2 to 3, respectively. The HR was mentioned only as a safety threshold in two studies^[17,23]^, which tolerated a 30 bpm increase considering resting HR. The exercise lasted from 10 to 20 minutes per session. All studies described a frequency of one to two sessions per day. Also, only one study^[22]^ reported fixed time progression for all patients. Noteworthy, the ACSM^[38]^ recommendations are for healthy individuals, and the guidelines on prescribing exercises for critically ill patients are uncertain. Nevertheless, few studies use more appropriate prescriptions, such as HR intensity control, a continuously monitored parameter in the ICU.

The phase II of CR presents an adequate exercise prescription^[14]^, whereas phase I does not have parameters well described in the literature. If exercise prescription items were well established and individualized in phase I, the benefits could be greater than those described in this review. The cycle ergometer exercises simplify intensity monitoring (*e.g.*, HR), which may be useful for prescribing exercise more individually. In addition, the portable cycle ergometer is low-cost, allowing an earlier mobilization at bedside^[39]^ and better results.

This study was the first systematic review and meta-analysis evaluating the effects of adding the cycle ergometer to the conventional physical therapy protocol after an open-heart surgery. The results showed that cycle ergometer exercise may improve functional capacity in a clinically relevant way and potentially reduce ICU stay. Future studies must include cycle ergometer exercise in the conventional physical therapy of this population, with better methodological quality, less risk of bias, and better control of exercise intensity, duration, and progression.

### Limitations

This review presents some limitations. Most studies had a high risk of bias for at least two items of the Cochrane tool. None of the studies blinded the patients; one^[22]^ did not blind evaluators. It is possible for this type of intervention to blind the evaluators; however, it is difficult to blind patients as they can perceive the exercise performed. The external validity of our results may also be compromised since only five studies were included, comprising a small sample, and most patients were men. In addition, Trevisan et al.^[17]^ had perioperative or PO complications as exclusion criteria, and Borges et al.^[22]^ excluded prolonged hospitalization time, limiting the extrapolation of the results for these patients. This may also have compromised the meta-analyses of ICU stay and total hospitalization time. Although the I^2^ values were low, the heterogeneity of the interventions and the evaluation period may have compromised the results of the meta-analyses. Lastly, the studies did not evaluate peripheral muscle strength and pulmonary complications, limiting further conclusions.

## CONCLUSION

In conclusion, cycle ergometer exercises added to a conventional physical therapy protocol improved the functional capacity of patients after open-heart surgery with no relevant effects on hospitalization time.

## Data Availability

The authors declare that data sharing is not applicable to this article as no new data were created or analyzed.

## Artificial Intelligence Usage

The authors declare that no artificial intelligence tools were used in the preparation of this article.
